# Thermoelectric magnetohydrodynamic effects on the crystal growth rate of undercooled Ni dendrites

**DOI:** 10.1098/rsta.2017.0206

**Published:** 2018-01-08

**Authors:** A. Kao, J. Gao, K. Pericleous

**Affiliations:** 1 Centre for Numerical Modelling and Process Analysis, University of Greenwich, Old Royal Naval College, Park Row, London SE10 9LS, UK; 2 Key Laboratory of Electromagnetic Processing of Materials (Ministry of Education), Northeastern University, Shenyang, 110819, People's Republic of China

**Keywords:** undercooled growth, magnetic field, thermoelectric magnetohydrodynamics, numerical modelling

## Abstract

In the undercooled solidification of pure metals, the dendrite tip velocity has been shown experimentally to have a strong dependence on the intensity of an external magnetic field, exhibiting several maxima and minima. In the experiments conducted in China, the undercooled solidification dynamics of pure Ni was studied using the glass fluxing method. Visual recordings of the progress of solidification are compared at different static fields up to 6 T. The introduction of microscopic convective transport through thermoelectric magnetohydrodynamics is a promising explanation for the observed changes of tip velocities. To address this problem, a purpose-built numerical code was used to solve the coupled equations representing the magnetohydrodynamic, thermal and solidification mechanisms. The underlying phenomena can be attributed to two competing flow fields, which were generated by orthogonal components of the magnetic field, parallel and transverse to the direction of growth. Their effects are either intensified or damped out with increasing magnetic field intensity, leading to the observed behaviour of the tip velocity. The results obtained reflect well the experimental findings.

This article is part of the theme issue ‘From atomistic interfaces to dendritic patterns’.

## 1. Introduction

The study of free undercooled growth of pure materials is key to understanding the fundamentals of solidification. Analytical models such as the Lipton, Kurz and Trivedi (LKT) theory [1,2] have provided a good match to experimental results, but are derived on the assumption of stagnant flow. Achieving such conditions in terrestrial experiments is difficult due to buoyancy and the influence of external forces other than gravity. For example, triggered undercooled solidification uses electromagnetic levitation [3,4] in order to avoid heterogeneous nucleation. In that situation, Lorentz forces generated by the AC levitation field act to counteract gravity. However, because the force is not uniformly distributed, relatively large bulk flow velocities can form in a molten and an undercooled liquid. Even in microgravity, surface tension (Marangoni) driven convection may affect the results [5,6]. To counter convection, a combination of AC and static magnetic fields has been employed on occasion, with the static field used to suppress flow [7,8]. In the glass fluxing experiments presented here, where the sample was supported by a holder inside the bore of a superconducting magnet, an AC field was initially used to melt and superheat the sample. With the AC field switched off, residual bulk flow velocities can persist past nucleation [9]. These velocities and the resulting convective heat transport in the sample are assumed to be a primary reason for the mismatch between existing theory and experiments [4]. The Alexandrov and Galenko (AG) theory [10,11] was developed to account for dendritic growth with convection. It is an extension of the LKT theory and assumes a component of the fluid velocity is incident to the growing tip. With this assumption, the AG theory has been successful in predicting the growth speed of succinonitrile dendrites in the presence of buoyancy-induced natural convection [12].

**Figure 1.**
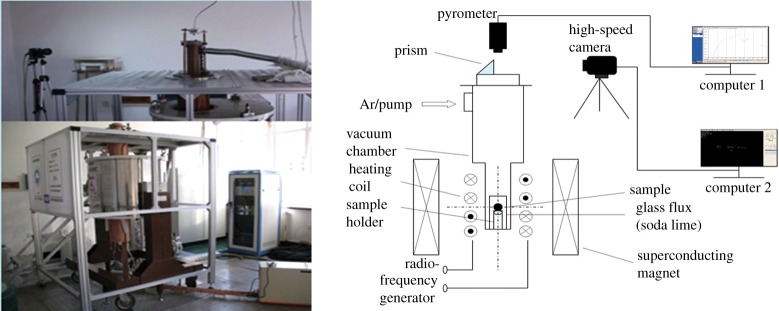
Experimental set-up of the glass fluxing method.

In previous work by the authors [12], experiments were conducted on 99.99% pure Ni. The sample was inductively heated and was undercooled by the glass fluxing treatment. During melting and undercooled solidification, a static magnetic field of intensity ranging from *B* = 0 T to *B* = 6 T was imposed on the sample, which was placed in the bore of a superconducting magnet. The sample was melted and solidified more than 20 times under each magnetic field intensity to acquire a wide spectrum of undercooling. Figure 1 shows the experimental set-up. During each cycle of melting and the following recalescence event, a pyrometer was used to measure the surface temperature of the sample and a high-speed camera was used to monitor and record the thermal front progression. The evolution of the thermal front along the surface of the sample can then be used to deduce the dendritic tip velocity in undercooled growth. Further details of the experimental set-up and procedures can be found here [12].

With increasing magnetic field intensity one would expect electromagnetic damping to remove all convection and the tip velocity dependence would level off with the magnetic field, conforming to a purely diffusion-driven system. Surprisingly, measurements of tip velocity of pure Ni [12] showed that a strong dependence on the magnetic field intensity remains even beyond values of *B* where the bulk velocity in the sample would be damped. One explanation offered was the introduction of additional convection, driven by the interaction of the external magnetic field with thermoelectric (TE) currents generated at the solid--liquid interface due to the Seebeck effect. Thermoelectric magnetohydrodynamics (TEMHD) is the term used to describe this phenomenon.

TEMHD was first described by Shercliff [13]. The formation of thermoelectric currents requires a spatial variation in the Seebeck coefficient, *S*, and a thermal gradient. In the context of undercooled growth, the Seebeck coefficient depends on temperature as well as phase change; essentially there is a discontinuity in the Seebeck coefficient between the solid and liquid phases. Owing to a relatively thin thermal boundary layer, dendrite tip curvature and kinetic undercooling, strong thermal gradients are an inherent part of undercooled growth. The resulting thermoelectric currents are, therefore, locally confined to the liquid–solid interface. In the presence of an external static magnetic field, the Lorentz force generated by the cross product of the magnetic field and current drives fluid flow within the thermal boundary. The localized convective transport can significantly alter the interface equilibrium conditions and the resulting tip velocity.

**Figure 2.**
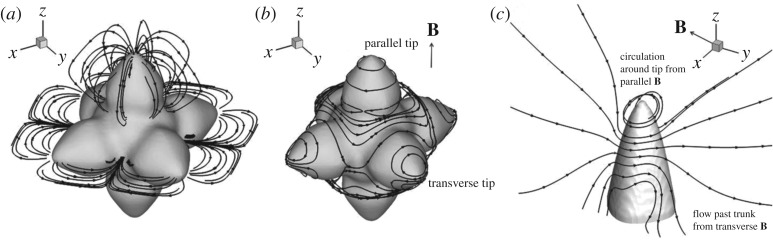
(*a*) Thermoelectric currents [12]. (*b*) TEMHD around an equiaxed set-up showing the flow field at the tips parallel and transverse to the magnetic field [12]. (*c*) Combined effect of the parallel and transverse magnetic fields illustrated by TEMHD around a single tip for a magnetic field oriented 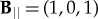.

Previous work by the authors has shown numerical examples of the TE current and TEMHD flow structure in undercooled growth [12,14]. Curvature variations along the dendrite surface lead to changes of temperature along the interface with cooler tips and warmer roots. TE currents then form closed paths between the tips and roots of a dendrite. This effect is shown in figure 2*a*, where, in this theoretical example, current emanates from the tips, passes along the dendrite and crosses the interface at the root. TEMHD is strongly dependent on the orientation of the magnetic field relative to the growth direction. This is highlighted in figure 2*b*, where a magnetic field oriented along the direction of growth interacts with the radial component of the current emanating from the tip, forming a force that drives a rotational flow around the tip. For a transverse magnetic field, TEMHD flow forms twin circulations around the tip that pass across the trunk of the dendrite and then over the tip. When the dendrite grows at an angle to the magnetic field, the resulting flow field is a combination of the flow fields from the parallel and transverse magnetic fields. Figure 2*c* shows this combination for circulation at the tip and flow past the trunk for a magnetic field 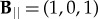. The figure highlights the dominant effect of each component of the magnetic field.

Based on the structure of these flow fields, the authors have qualitatively examined the underlying mechanism for the tip velocity dependence on the magnetic field in [15]. Application of the AG theory suggests that the TEMHD fluid velocities should be of the order of the tip velocity itself to cause any significant changes. However, due to the unidirectional flow assumption of the AG theory, it is not clear which aspect of the TEMHD flow structure this flow represents, or indeed if the AG theory is applicable in these conditions. To understand fully the underlying mechanism, the focus of this paper is to directly model the time evolution of the tip, solve the complete coupled governing equations for solidification, thermoelectricity and fluid flow, and so predict the equilibrium tip velocity.

## 2. Numerical modelling

### (a) Assumptions

With the introduction of the magnetic field, the system becomes increasingly complex, with many free parameters and their inter-dependences. A key parameter is the relative orientation of the magnetic field to dendritic growth, which in itself may be a function of the rather random heterogeneous nucleation location caused by the catalytic impurities at the melt--glass interface. Nevertheless, through the data fitting process, the presented magnetic field dependence would reflect the dominant orientation. Based on observations, the solid front evolution is typically tilted relative to the magnetic field. A statistical analysis of the nucleation events and the corresponding growth orientation is worthy of investigation, but it is outside the scope of the magnetic field intensity dependence focus of this paper. Instead, based on observations and looking at the geometry of the set-up in figure 1, we assume that the dendrites grow at a 45° angle relative to the magnetic field.

The other important parameter affecting growth kinetics is undercooling. The experimental results have highlighted that TEMHD effects are most significant in the low undercooling region. In this region, surface energy effects dominate interfacial undercooling. Therefore, the effects of kinetic undercooling are neglected and the model is most applicable for low undercoolings. Thus, by constraining the orientation and undercooling, only the magnetic field intensity dependence remains as a key parameter.

### (b) Governing equations

Using an enthalpy approach [14] based on the work of Voller [16], solidification can be described by the sum of sensible latent heats,
2.1
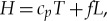

where *H* ( J m^−3^) is the volumetric enthalpy, *c_p_* ( J K^−1^ m^−3^) is the volumetric specific heat, *T* (K) is the temperature, *f* is the liquid fraction and *L* ( J m^−3^) is the volumetric latent heat. In the absence of kinetic undercooling, the Gibbs–Thompson condition is governed by surface energy, and the interfacial temperature *T^i^* is given by
2.2
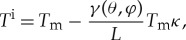

where *T*_m_ (K) is the melting temperature, 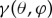 ( J m^−2^) is the surface energy and 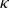 (m^−1^) the curvature. Convective transport of heat is given by
2.3
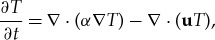

where 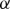 (m^2^ s^−1^) is the thermal diffusivity and **u** (m s^−1^) is the flow velocity. The current density, **J** (A m^−2^) is given by a generalized form of Ohm's law including an extra term accounting for thermoelectric currents,
2.4
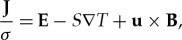

where *σ* (S m^−1^) is the electrical conductivity, **E** (V m^−1^) the electric field, *S* (V K^−1^) the Seebeck coefficient and **B** (T) the static magnetic field. Conservation of charge gives
2.5
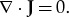


Owing to the short length scales encountered on the scale of a dendrite, the Reynolds number is assumed to be small and convection is described by the Stokes flow regime,
2.6
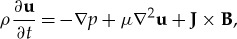

where *ρ* (kg m^−3^) is the density, *p* (N m^−2^) is the pressure, *μ* (Pa s) is the dynamic viscosity and 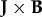 is the Lorentz force. Conservation of mass for an incompressible flow gives
2.7
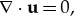

completing the physical set of governing equations.

The numerical code ‘Thermoelectric Solidification Algorithm' (TESA) has been developed to couple the three key physical phenomena. TESA uses a dimensionless form to solve the set of equations. The base SI unit scaling factors are defined by
2.8
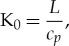

2.9
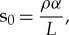

2.10
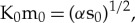

2.11
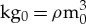

2.12
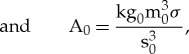

for kelvin, second, metre, kilogram and amp, respectively. The dimensionless density 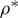, thermal diffusivity 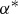 and electrical conductivity 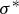 are all equal to 1.

### (c) Problem set-up

The computational domain is a cuboid populated with a uniform Cartesian mesh with cell dimensions of 256 × 256 × 128. The boundary conditions of the problem for all faces are
2.13
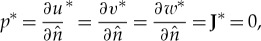

where 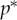, 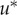, 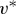, 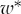, J* and 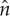 represent the dimensionless pressure, three velocity components, current density and the normal vector to the face, respectively. For dimensionless temperature 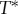 all faces except for the low face have
2.14
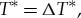

where 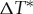 is the dimensionless undercooling, while at the low face
2.15
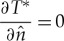

applies. All of the boundaries except for the low face of the domain represent far-field bulk conditions. Initially a solid nucleus is placed at the centre of the low face. As solidification progresses, TE currents form and TEMHD flow develops. Convective transport alters the thermal field, modifying the thermal equilibrium at the interface, which in turn alters **J** and TEMHD. It is assumed that after some time the system reaches a quasi-steady state, where the tip velocity becomes constant, in analogy to the microscopic solvability theory [17]. To capture the tip evolution as it falls into this equilibrium, a moving mesh technique is adopted, where at equilibrium conditions the tip velocity equals the moving mesh velocity. From a computational perspective, the mesh moves when the solidification front reaches a layer of cells half way up the domain.

## 3. Results and discussion

The numerical results present a parametric study of magnetic field intensity that captures the time evolution of the tip until the equilibrium velocity is found. Constant dimensionless parameters are used in all cases, as given in table 1. These dimensionless values represent characteristic material properties for metal solidification. The numerical results then represent a family of solutions that all exhibit the same general trend. Corresponding dimensioned values based on the material properties of Ni are also given in table 1.

**Table 1.** Parameters.

variable	symbol	dimensionless value	dimensioned value
cell size	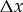	25.6	5.49 × 10^−7^ m
density	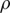	1	8.01 × 10^3^ kg m^−3^
dynamic viscosity	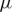	6.43 × 10^−2^	6.00 × 10^−3^ Pa s
latent heat	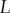	1	2.35 × 10^9^ J m^−3^
specific heat	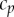	1	5.28 × 10^6^ J m^−3^
electrical conductivity	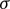	1	2.08 × 10^6^ S m^−1^
thermal diffusivity	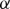	1	1.16 × 10^−5^ m^2^ s^−1^
Seebeck coefficient solid	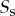	−0.098	−2.52 × 10^−5^ V K^−1^
Seebeck coefficient liquid	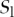	−0.294	−7.56 × 10^−5^ V K^−1^

**Figure 3.**
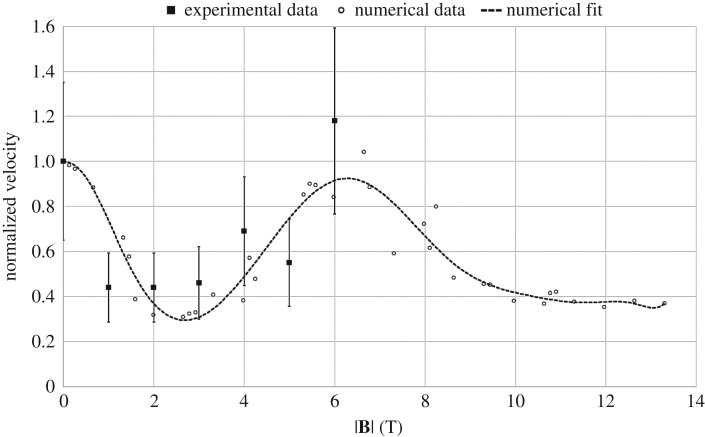
Comparison of normalized tip velocity between experimental and numerical results.

To compare the numerical results to experimental data, the steady-state velocity is first normalized against the numerical 0 T case with no fluid flow. A high 12th-order polynomial was then fitted to the numerical data. Then a least-squares regression of this numerical trend was applied to the experimental data, providing a qualitative comparison of the predicted and observed trends. Figure 3 shows the comparison of the numerical trend to experimental data of 60 K undercooling of 99.99% pure Ni [9,12,15]. The 60 K undercooling corresponds to a dimensionless thermal undercooling 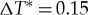 used in the numerical calculations. Although the comparison is qualitative due to the various assumptions used in creating a tractable model and uncertainty in the value of the Seebeck coefficient, the main trends are captured reasonably well. The results show that there are three magnetic field intensity regimes: a low magnetic field of 0–2.7 T, where the tip velocity decreases; a moderate magnetic field of 2.7–6.7 T, where the tip velocity recovers; and a high magnetic field greater than 6.7 T, where the tip velocity decreases and plateaus. There are two critical magnetic fields, where the tip velocity reaches a minimum or maximum, which, by these definitions, represent the transition from the low to moderate magnetic field regime and from the moderate to high magnetic field regime, respectively.

**Figure 4.**
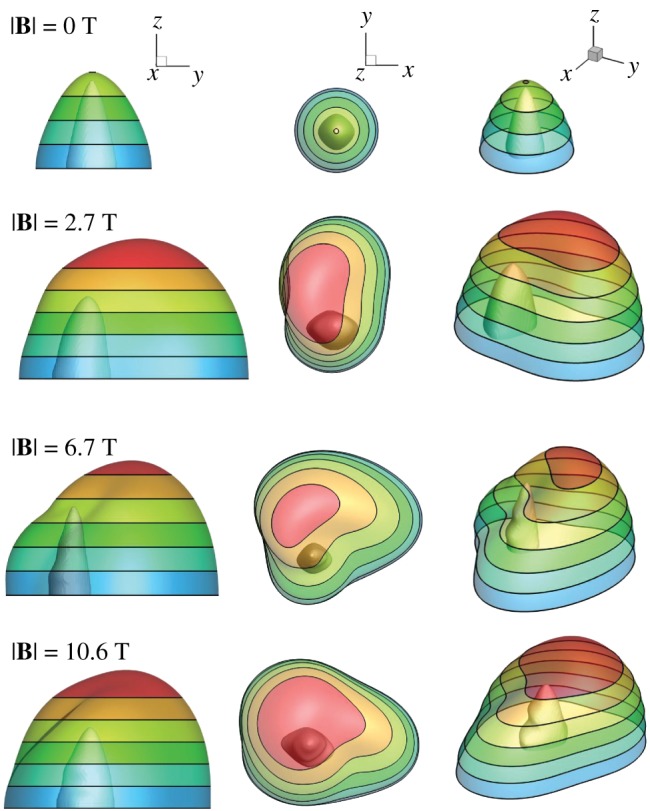
Tip morphology and thermal boundary layer, 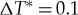 for increasing magnetic field. The thermal isosurfaces are coloured by the *z* distance for clarity in highlighting the boundary layer shape.

By analysing the effect of TEMHD on the thermal boundary layer, the underlying mechanism that gives rise to these trends can be identified. The numerical results suggest that the mechanism is a combination of the transverse and parallel flow fields highlighted in figure 2*c*. Figure 4 shows the steady-state three-dimensional solid structure and surrounding thermal boundary layer at 0 T, the transition from low to moderate magnetic field at 2.7 T, the transition from moderate to high magnetic field at 6.7 T, and for a high magnetic field at 10.6 T. At 0 T, the tip is essentially a paraboloid; thermal transport is diffusive and approximately equal in all directions, and both the tip and the thermal boundary layer remain axisymmetric.

As the magnetic field increases through the low magnetic field regime, the transverse field effects become prominent, represented in figure 4 at 2.7 T. Heat is transported across and up the trunk of the dendrite towards the tip, generating an extended thermal boundary layer orthogonal to both the growth direction and the transverse magnetic field. The increase in temperature local to the tip reduces the free energy and slows the rate of solidification. This part of the mechanism is responsible for the reduction in tip velocity between 0 and 2.7 T in figure 3.

With further increase in the magnetic field intensity, thermal transport becomes a combination of both the transverse and parallel field effects. Heat is still ejected tangentially and towards the tip by flow from the transverse magnetic field component, but flow from the parallel component introduces rotation in the thermal boundary layer. This combined effect introduces swirl in the flow field, where cooler liquid is directed onto the tip due to the breaking of axisymmetry. Consequently, the tip velocity recovers between 2.7 and 6.7 T, as shown in figure 3. The thermal boundary layer where this effect reaches the critical point is represented by the 6.7 T result in figure 4.

Finally, with further increase in the magnetic field intensity, electromagnetic damping becomes dominant. The transverse field effect diminishes, which can be seen in figure 4 by a partial return to axisymmetry. The rotational flow from the parallel field becomes increasingly confined to the interface close to the tip. Convective transport from the rotating flow will act to homogenize the temperature close to the tip and will dominate over any underlying surface energy anisotropy. The resultant effect is a coarsening of the tip and a corresponding reduction in the tip velocity. This is shown for a 10.6 T field in figure 4. Unfortunately, the experimental results for Ni do not extend beyond 6 T. However, this magnetic damping effect has been observed to occur at lower magnetic fields in Pd experiments, as yet unpublished.

The relative effects of the transverse and parallel components of the magnetic field are determined by the balance of the driving thermoelectric force and the damping electromagnetic force. There is a direct analogy between the findings of this work and those conducted by Kao [18] in directional solidification of alloys. The flow fields for both the parallel and transverse magnetic fields exhibit similar features to those explored in this work. Kao [18] showed that critical magnetic field intensities exist for each orientation of the magnetic field. These critical magnetic fields were based on characteristic length scales associated with the geometry of the problem, for example, tip radius and primary arm spacing. One of the key findings was that the transverse field component has a lower critical field than that of the parallel component. A similar observation is made here, where the transverse magnetic field effects dominate the low magnetic field regime, and the parallel magnetic field effects dominate the high magnetic field regime. However, determining the characteristic length scales associated with the critical magnetic field is more complex in undercooled growth. Physically, the characteristic length scale should represent the viscous boundary layer, which is governed by the length scale of the driving thermoelectric force and hence the length scale of **J**, with **J** intimately coupled to the surface temperature and dendrite morphology. The parallel component of the field interacts with radial currents emanating from the tip and the transverse component interacts with currents passing down the trunk of the dendrite. By this hypothesis, the characteristic length scales for the critical magnetic fields should be functionally dependent on the tip radius and trunk radius for the parallel and transverse components, respectively. Where the tip is smaller than the trunk, this would give rise to a higher critical magnetic field in the parallel case. However, it is not yet clear what these functional dependences are and further investigation is necessary both theoretically and experimentally to see if the trend described here is general to all thermoelectrically active metals. Conducting experiments on other materials with varying thermo-physical properties and also solidification properties such as morphology will help to further elucidate the underlying mechanism and its dependence.

## 4. Conclusion

The effect of TEMHD on dendritic growth in undercooled Ni was examined at various intensities of imposed static magnetic field. A purpose-built numerical code was used to compare numerical predictions with experiments, in an effort to explain the driving mechanism responsible for observed variations in dendrite tip velocity, which cannot be explained by existing theories such as the LKT theory, as they do not consider the TEMHD phenomenon. The numerical results highlight a series of regimes depending on magnetic field intensity that characterize experimental observations. The computations have shown that in the low magnetic field regime, flow generated by the transverse component of the field is dominant, giving rise to thermal pile-up ahead of the tip, slowing growth. With increasing magnetic field, the flow generated by the parallel component introduces a rotating flow around the tip, which re-introduces bulk temperature to the tip, restoring the tip velocity due to asymmetry in the thermal boundary layer. In the high magnetic field regime, electromagnetic damping suppresses the transverse flow field, while the rotating flow field associated with the parallel component becomes confined to the tip, resulting in a coarsening effect, reducing the tip velocity. Further studies of this effect are continuing with a series of pure metals and alloys.

## Data accessibility

The datasets supporting this article have been uploaded to the University of Greenwich's repository, available from gala.gre.ac.uk.
